# Hypoxia-Inducible Factor as an Angiogenic Master Switch

**DOI:** 10.3389/fped.2015.00033

**Published:** 2015-04-24

**Authors:** Takuya Hashimoto, Futoshi Shibasaki

**Affiliations:** ^1^Department of Surgery, Yale University School of Medicine, New Haven, CT, USA; ^2^Division of Vascular Surgery, Department of Surgery, Graduate School of Medicine, The University of Tokyo, Tokyo, Japan; ^3^Department of Molecular Medical Research, Tokyo Metropolitan Institute of Medical Science, Tokyo, Japan

**Keywords:** HIF-1α, HIF-2α, pVHL, Int6/eIF3e, angiogenesis, peripheral arterial disease, collateral vessel

## Abstract

Hypoxia-inducible factors (HIFs) regulate the transcription of genes that mediate the response to hypoxia. HIFs are constantly expressed and degraded under normoxia, but stabilized under hypoxia. HIFs have been widely studied in physiological and pathological conditions and have been shown to contribute to the pathogenesis of various vascular diseases. In clinical settings, the HIF pathway has been studied for its role in inhibiting carcinogenesis. HIFs might also play a protective role in the pathology of ischemic diseases. Clinical trials of therapeutic angiogenesis after the administration of a single growth factor have yielded unsatisfactory or controversial results, possibly because the coordinated activity of different HIF-induced factors is necessary to induce mature vessel formation. Thus, manipulation of HIF activity to simultaneously induce a spectrum of angiogenic factors offers a superior strategy for therapeutic angiogenesis. Because HIF-2α plays an essential role in vascular remodeling, manipulation of HIF-2α is a promising approach to the treatment of ischemic diseases caused by arterial obstruction, where insufficient development of collateral vessels impedes effective therapy. Eukaryotic initiation factor 3 subunit e (eIF3e)/INT6 interacts specifically with HIF-2α and induces the proteasome inhibitor-sensitive degradation of HIF-2α, independent of hypoxia and von Hippel-Lindau protein. Treatment with eIF3e/INT6 siRNA stabilizes HIF-2α activity even under normoxic conditions and induces the expression of several angiogenic factors, at levels sufficient to produce functional arteries and veins *in vivo*. We have demonstrated that administration of eIF3e/INT6 siRNA to ischemic limbs or cold-injured brains reduces ischemic damage in animal models. This review summarizes the current understanding of the relationship between HIFs and vascular diseases. We also discuss novel oxygen-independent regulatory proteins that bind HIF-α and the implications of a new method for therapeutic angiogenesis using HIF stabilizers.

## Introduction

Oxygen is essential for eukaryotic life, and changes in oxygen availability can lead to cell and organ dysfunction. To ensure adequate oxygen delivery, metazoans have developed complex and elaborate systems that respond to hypoxia, as seen in the circulatory and respiratory systems in mammals. Cellular responses to hypoxia are mainly regulated by the activation of transcription factors called hypoxia-inducible factors (HIFs) (1, 2). HIFs affect hypoxia and stress response signaling pathways that influence development, metabolism, inflammation, and circulatory and respiratory physiology (1–4).

Structurally, HIFs are heterodimers comprised an oxygen-regulated HIF-1α or HIF-2α subunit and a constitutively expressed HIF-1β subunit. The HIF-1α and HIF-2α subtypes have high identity in their functional domains (5), and their expression and transcriptional activity are regulated by the hydroxylation of specific proline and asparagine residues (6). On the other hand, HIF-1α and HIF-2α have distinct expression patterns. HIF-1α is ubiquitously expressed in all mammalian tissues and cell types (7). In contrast, HIF-2α expression is restricted to specific cell types, including endothelial cells, but is not confined to the vasculature, with a distinct distribution in cells and tissues (8). Moreover, the degree of hypoxia needed to induce each subtype differs. HIF-2α is induced at higher oxygen concentrations and for longer durations than HIF-1α (9). These findings indicate that the two subtypes have complementary functions in the coordinated transcriptional response to hypoxia/stress.

Although HIFs were originally identified as oxygen-dependent transcription factors, recent studies have provided evidence for the hypoxia-independent regulation of HIFs, as described later. The differences in the mechanisms that regulate HIF-1α and HIF-2α provide further evidence for the proteins’ distinct but coordinated functions.

Hypoxia-inducible factors are disrupted in cancer and disorders affecting the circulatory system. Most human cancers exhibit increased levels of HIF subtypes (10, 11), and HIF expression levels correlate with mortality (12). The hypoxic environment within solid tumors and various genetic alterations contribute to alterations in HIF activity. HIF target genes play important roles in all aspects of cancer biology, including angiogenesis, cell survival, metabolism, invasion, and metastasis (13, 14). Dissecting the HIF pathway is of major clinical significance because the hypoxic response correlates with tumor progression and resistance to therapy (15). Several anti-cancer treatment strategies targeting the HIF pathway have already been applied in clinical settings (16–18). However, the inconclusive results of the clinical trials suggest that further analysis of HIF biology and improved selection of patient subpopulations are needed.

Hypoxia-inducible factors are also associated with many diseases in the circulatory system. Given that HIF pathways have evolved to maintain oxygen homeostasis, it is no wonder that their disruption causes pathophysiology in the circulatory system. One example is ischemic disease due to arterial obstruction, where an impaired response to ischemia in diabetes or aging is a critical risk factor. Therefore, controlling angiogenesis and arteriogenesis by modulating the HIF pathway could be a valuable strategy in patients with ischemic diseases. Clinical studies have only just begun, and several studies using gene or protein delivery to stabilize HIFs have failed to show efficacy (19–21). Methodology, including the choice of HIF subtype, the method of drug delivery, and the combined use of cell therapy, will be key factors in the success of HIF-modulating therapy.

In this review, we summarize the current understanding of the relationship between HIFs and diseases in the circulatory system. We also focus on binding proteins that regulate HIFs and the implications of new methods for therapeutic angiogenesis that use HIF stabilizers.

### Basic mechanism for the response of HIFs to hypoxia

Hypoxia-inducible factors are DNA-binding transcription factors that associate with specific nuclear cofactors under hypoxia. Heterodimeric proteins, HIFs consist of an oxygen-regulated subunit (HIF-1α or HIF-2α) and a constitutively expressed HIF-1β subunit [aryl hydrocarbon receptor nuclear translocator, ARNT1/(HIF-1β) or ARNT2/(HIF-2β)] (22–24). HIFs have a characteristic Per–ARNT–Sim (PAS) domain and belong to the basic helix–loop–helix (bHLH) transcription factor superfamily (24). In humans, three genes encode distinct HIF-α isoforms: *HIF1A*, encoding HIF-1α; *EPAS1* or *HIF2A*, encoding HIF-2α, and *HIF3A*, encoding multiple HIF-3α splice variants (24–26). HIF-1α is ubiquitously expressed, whereas HIF-2α (originally named endothelial PAS domain protein-1 (EPAS-1) is abundant in endothelial cells and some highly vascularized tissues (5). HIF-1α and HIF-2α have 48% amino acid identity and similar protein structures. Their bHLH domains and PAS regions are 83 and 70% identical, respectively. Furthermore, the oxygen-dependent degradation domains (ODD) in the two HIF-α subunits, including the two critical proline residues, exhibit a high degree of homology (27). Prolyl-hydroxylase domain (PHD) 2 binds to both HIF-α subtypes and catalyzes the hydroxylation of Pro-402 and Pro-564 in HIF-1α and Pro-405 and Pro-531 in HIF-2α. Factor-inhibiting HIF (FIH)-1 also binds to both HIF-α subtypes and catalyzes the hydroxylation of Asn-803 in HIF-1α and Asn-851 in HIF-2α. However, HIF-1α and HIF-2α play distinct, non-redundant biological roles because of their different expression patterns and binding partners (28). HIF-1α and HIF-2α regulate their target genes by binding to hypoxia-responsive elements (HREs). Many target genes are transactivated by either HIF-1α or HIF-2α, and some genes are upregulated by both.

The main sensors of the hypoxic condition are the HIF-α subunits. HIF-1α and HIF-2α each contain an ODD and two transactivation domains, an N-terminal transactivation domain (N-TAD) and a C-terminal transactivation domain (C-TAD) (26, 29–36). However, HIF-3α does not have a C-TAD, suggesting that its function is regulated in a simpler manner (37). The stability of the HIF-α proteins is negatively regulated by PHD-dependent hydroxylation in a post-translational manner. Under normoxia, HIF-α proteins are hydroxylated on at least one of two conserved proline residues within the ODD by PHD-containing enzymes (38) and rapidly degraded via the von Hippel-Lindau (pVHL)-ubiquitin-proteasome pathway (38–40). The key HIF-1α destabilizing enzyme in normoxia is PHD2 (41). Chelators of cobalt and iron ions such as desferrioxamine, which mimic hypoxia, inhibit hydroxylases by displacing Fe(II) from the ferroprotein oxygen sensor (38).

Another hydroxylase domain-containing protein, named FIH, participates in the negative regulation of HIF-α by hydroxylating Asn-803 in the C-TAD in the presence of oxygen, thereby inhibiting the interactions between HIF-α and transcriptional co-activators (42). Dayan et al. reported that the N-TAD and C-TAD have distinct functions. FIH controlled a spectrum of gene expression, consistent with further fine-tuning of HIF-1α regulation, by binding the C-TAD in severe hypoxia, independent of PHD binding to the N-TAD in intermediate hypoxia (37).

When cells are exposed to hypoxic conditions, the oxygen-requiring hydroxylation process is prevented, and HIF-α subtypes escape proteasomal degradation. The HIF-α subtypes then dimerize with HIF-1β and associate with transcriptional co-activators. The transcriptional complex subsequently recognizes HREs in various hypoxia-responsive genes, resulting in physiological adaptation to hypoxia. Oxygen depletion also reduces FIH-mediated Asn-803 hydroxylation, allowing HIF-α to interact with the transcriptional co-activators p300/CREB-binding protein (CBP) (43). The transcriptional complex recognizes the HREs in downstream responsive genes, leading to the adaptive response to hypoxic stress. Of note, stimuli other than hypoxia, such as nitric oxide and reactive oxygen species, can also activate HIFs (2).

### Genes downstream of HIFs

Hypoxia-inducible factor-1α was originally identified as a protein whose binding to the HRE in the human erythropoietin (*EPO*) gene was required for transcriptional activation in response to a reduced cellular O_2_ concentration (22). EPO increases the blood O_2_-carrying capacity by stimulating erythropoiesis. Other HIF downstream genes regulate processes such as glucose uptake, glycolysis, angiogenesis, vascular remodeling, extracellular matrix metabolism, inflammation, cell proliferation, apoptosis, autophagy, migration and invasion, DNA damage responses, and survival (3, 12, 13). The encoded proteins, which play roles in systemic, tissue, or intracellular O_2_ homeostasis, include vascular endothelial growth factor (VEGF) (44), which mediates vascularization, and inducible nitric oxide synthase (iNOS) (45) and heme oxygenase 1 (HO1) (46), which modulate vascular tone. A central adaptation to hypoxia is the shift toward anaerobic glycolysis. HIF-1α guides this shift by promoting the expression of glucose transporters and glycolytic enzymes (1, 47–49). Under hypoxia, HIF-1 mediates a transition from oxidative to glycolytic metabolism by regulating genes such as pyruvate dehydrogenase kinase 1 (50, 51), lactate dehydrogenase A (52), and BNIP3/BNIP3L, which mediate mitochondrial autophagy (53–55).

### Activation of HIF-1α transcriptional activity by histone deacetylase 7

Using the yeast two-hybrid method, Kato et al. identified a novel transcriptional activator of HIF-1α, histone deacetylase 7 (HDAC7) (56). HDAC7 is a transcriptional repressor that belongs to the mammalian class II HDAC family, whose members include HDAC4, HDAC5, HDAC6, HDAC7, HDAC9, and HDAC10. HDAC4, HDAC5, and HDAC7 contain a highly conserved catalytic domain (HDAC domain) in the C-terminal region (57, 58). However, the N-terminal region and C-terminal tail of HDAC7 and the corresponding regions of HDAC4 and HDAC5 are less homologous (59, 60). The catalytic domain of HDAC7 interacts with the inhibitory domain (ID) of HIF-1α in both normoxia and hypoxia. Kato et al. also found that the regions containing amino acids 735–785 in HIF-1α and amino acids 669–952 in HDAC7 were the minimum contact sites required for the interaction. HDAC7 bound solely to HIF-1α, among the HIF-α isoforms, while HIF-1α only interacted with HDAC7 in the class II HDAC family. HIF-2α and HIF-3α do not contain the ID found in HIF-1α. Therefore, the ID domain likely plays an important role in regulating the transcriptional activity of HIF-1α by mediating the interaction with HDAC7. Although HDAC7 was predominantly localized to the cytoplasm at normal oxygen concentrations, HDAC7 co-translocated to the nucleus with HIF-1α under hypoxia. Thus, HDAC7 forms a complex with HIF-1α and CBP/p300 in the nucleus under hypoxic condition, leading to enhanced transcription of HIF-1α target genes (*VEGF* and *Glut-1*). Conversely, HDAC4 and HDAC5 did not bind HIF-1α (56). Immunoprecipitation experiments suggested that HIF-1α, HDAC7, and p300 formed a complex in the nucleus. The binding of HDAC7 to HIF-1α might lead to a conformational change within the ID of HIF-1α that facilitates binding to co-activators such as CBP/p300 and increases transcriptional activity under hypoxia.

### Differences in the functions of the HIF subtypes

Hypoxia-inducible factor-2α, a paralog of HIF-1α, is also regulated by prolyl and asparaginyl hydroxylation in vertebrates (55). Unlike HIF-1α, HIF-2α is mainly expressed in vascular endothelial cells. Therefore, HIF-2α is thought to regulate endothelial-specific genes and have functions different from those of HIF-1α. Efforts to distinguish the roles of HIF-1α and HIF-2α are ongoing. Like HIF-1α, HIF-2α is also stabilized during hypoxia; HIF-2α forms a heterodimer with ARNT and transactivates the promoters of genes such as *VEGF* and *EPO*. Although O_2_ regulates the stability of both proteins in a similar manner, HIF-2α was stabilized and localized to the nucleus of bovine arterial endothelial cells even under normoxia (35, 61), suggesting that the subtypes have different roles that depend on the degree of oxygen availability.

*Hif-1*α^−/−^ mice exhibit mid-gestation lethality due to severe cardiac malformations, blood vessel defects, and impaired erythropoiesis (1, 62), indicating that major components of the circulatory system are dependent on HIF-1 for normal development. On the other hand, *Hif-2*α^−/−^ mice manifest defective vascular remodeling during embryonic development (63), as well as defective catecholamine homeostasis (64), fetal lung maturation, and hematopoietic cell production (65). Semenza proposed that both HIF-1 and HIF-2 have important roles in circulatory system development, although the appearance of the circulatory system and HIF-2α are associated in vertebrate evolution (55).

Although the structures of HIF-1α and HIF-2α are similar, the proteins activate distinct target genes. Whereas some genes are robustly activated by both HIF-1 and HIF-2, other genes are preferentially activated by one factor (48, 49). DNA microarray analysis in renal cell carcinoma cells that exclusively expressed HIF-2α (but not HIF-1α) (48) showed that a number of hypoxia-inducible genes were expressed, including *CITED2*, a putative negative regulator of HIF-1α activity (66, 67).

The C-TAD and N-TAD confer HIF target gene specificity by interacting with additional transcriptional cofactors (49). The C-TADs in HIF-1α and HIF-2α are highly homologous; and the domain promotes the expression of genes commonly regulated by HIF-1α and HIF-2α. The N-TADs are less homologous and are important for target gene specificity. The HIF-3α splice variants are homologous to HIF-1α and HIF-2α, but lack the C-TAD or N-TAD. Therefore, HIF-3α cannot induce gene expression, and it is thought to have an inhibitory effect on HIF-1α- and HIF-2α-induced gene expression (25, 68). Although HIF-1α and HIF-2α respond to similar cell stimuli, they often control different pathways. The degree to which the roles of HIF-1α and HIF-2α overlap or compensate remains a question. For example, hypoxic induction of HIF-1α target genes is attenuated in HIF-1α-deficient endothelial cells, suggesting that HIF-2α or other hypoxia-induced factors cannot sufficiently compensate for the loss of HIF-1α.

### miRNA regulation of HIF mRNA

Recent studies have focused on the induction of miRNAs that positively or negatively affect the transcription of specific mRNAs. miRNAs are a class of endogenous tiny RNAs that inhibit translation or promote RNA degradation by forming a duplex within the untranslated region of mRNAs. miRNAs play an important role in a wide range of cellular processes by fine-tuning gene expression (69, 70). Bruning et al. reported that miR-155 contributes to the isoform-specific downregulation of HIF-1α activity in cells exposed to prolonged hypoxia (71). Bartoszewska et al. showed that HIF-1 is in a negative regulatory loop with miR-429 (72). While the activity of stabilized HIF-1 increases miR-429 expression, miR-429 attenuates HIF-1 activity by decreasing HIF-1α mRNA levels during the early stages of hypoxia in endothelial cells. Poitz et al. showed that miR-17 and miR-20a target HIF-1α and HIF-2α during the adaptation of macrophages to hypoxia (73). The mechanisms by which miRNAs regulate HIFs require further investigation.

## Oxygen-Independent Regulation of HIFs

As described above, the regulation of HIFs is dependent on the oxygen concentration. Although HIF-2α is not strictly regulated, the stability of HIF-1α is completely dependent on the oxygen concentration. The key regulator of HIF-α is pVHL, which mediates the oxygen-dependent, proteasomal degradation of HIF-α in normoxia by binding to hydroxylated proline residues (Pro-402 and Pro-564 in human HIF-1α) and promoting ubiquitin binding. Three new binding factors regulate oxygen-independent regulation: hypoxia-associated factor (HAF), small ubiquitin-related modifier (SUMO)-specific protease 1, and Int6/eukaryotic initiation factor (eIF) 3e, a translation initiation factor. These factors promote HIF degradation in a similar manner by binding directly to HIFs and triggering ubiquitin-proteasome activation. However, SUMO-specific protease 1 and HAF are specific to HIF-1α, whereas Int6 is specific to HIF-2α. We describe the three hypoxia-independent regulators of HIF-α in the sections that follow.

### HIF-1α-specific regulation

#### HAF in HIF-1α Degradation

Hypoxia-associated factor is an E3 ligase for HIF-1α that mediates the subtype-specific proteasomal degradation of HIF-1α in an oxygen- and pVHL-independent mechanism (74). HAF, also known as SART1_800_ (*s*quamous cell carcinoma *a*ntigen *r*ecognized by *T* cells), was originally identified as a nuclear protein expressed in proliferating cells (75).

Hypoxia-associated factor is overexpressed in a variety of tumors. Its levels decrease during acute hypoxia, but increase following prolonged hypoxia. HAF binds to the ODD in HIF-1α and induces ubiquitination. In contrast, in HIF-2α, HAF binds to the region between the N-TAD and C-TAD and increases HIF-2α activation, thereby inducing a switch from HIF-1α- to HIF-2α-dependent response to chronic hypoxia (28, 74). The process activates genes involved in invasion, such as matrix metalloproteinase (MMP)-9, PAI-1, and the stem cell factor OCT-3/4, resulting in more aggressive growth of tumors under prolonged hypoxia (28). Guan et al. reported that activation of the NF-κB pathway drives the HAF-mediated switch from HIF-1α to HIF-2α in bladder cancer cells (76). Koh et al. recently described the role of SUMOylation (discussed later) in HAF activation (77). In clear-cell renal cell carcinoma (CRCC), hypoxia induced HAF SUMOylation without affecting HAF-mediated HIF-1α degradation. On the other hand, HAF overexpression in a mouse model increased CRCC growth and metastasis. Koh et al. also confirmed that HAF overexpression was associated with poor prognosis in a clinical setting. The findings indicate that HAF acts as a specific mediator of HIF-2 activation that is critical for CRCC development and morbidity.

#### Role of SUMO-Specific Protease 1 (SENP1) in HIF-1α Stability

Small ubiquitin-related modifiers are small proteins that share low sequence identity but high structural similarity with ubiquitin (78). SUMO post-translationally modifies many proteins and regulates protein localization and activity. Thus, SUMOylation affects diverse cellular functions, including transcription (79), nuclear translocation (80), the stress response (81), and chromatin structure (82). SUMOylation is catalyzed by SUMO-specific ligases and reversed by SUMO-specific proteases (SENPs). Cheng et al. generated a SENP1 knockout mouse, in which sumoylated HIF-1α was unstable (83). SENP1 knockout embryos exhibited severe anemia stemming from deficient Epo production that was lethal during mid-gestation. Further experiments showed that SENP1 controlled Epo production by regulating the stability of HIF-1α. The authors identified a role for the E3 ubiquitin ligase VHL in sumoylated HIF-1α degradation. Hypoxia induced the SUMOylation of HIF-1α, which led to the hydroxylation-independent binding and subsequent degradation of HIF-1α by the pVHL–E3 ligase complex (83). The results indicate that SENP1 is essential for the stabilization of HIF-1α during hypoxia.

### HIF-2α-specific regulation

#### Int6/Eukaryotic Initiation Factor 3 Subunit e in HIF-2α Regulation

Eukaryotic initiation factor 3 is a highly complex, multiprotein assembly that regulates translation initiation by orchestrating the formation of 43S–48S preinitiation complexes (84). The highly conserved *eIF3e* gene has been described in yeast and mammals. The gene encoding eIF3e, also called Int6, was first identified as a tumor suppressor gene based on frequent integration of mouse mammary tumor virus (MMTV) (85). MMTV insertion into mouse Int6-coding DNA sequences appears to create a C-terminal truncated protein, which functions as a dominant-negative mutant. Overexpression of the truncated protein transforms cells in culture, and injection of the transformed cells into nude mice induces tumor formation (86, 87). eIF3e has also been characterized in rabbits (88), *Schizosaccharomyces pombe* (89–91), and *Arabidopsis thaliana* (92, 93).

**Figure 1:**
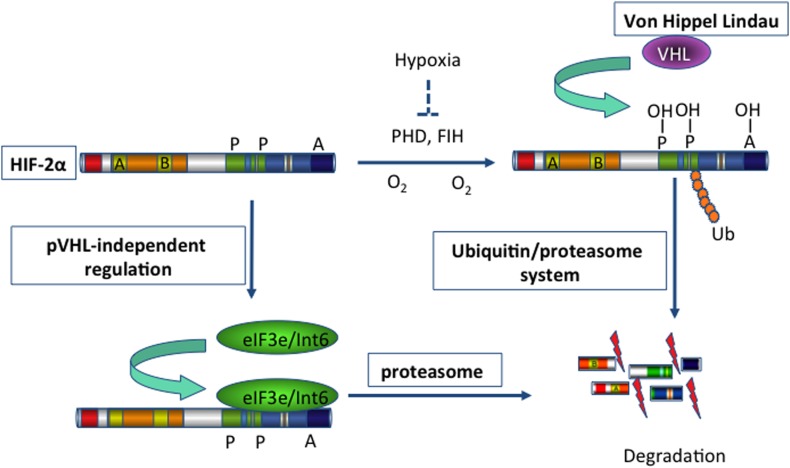
**Schema illustrating the degradation of HIF-2α**. Hypoxia-inducible factor 2 (HIF-2) activates gene transcription in response to hypoxia. Under normoxic conditions (blue arrows), HIF-2α is hydroxylated on proline residues 496 and 542 by a prolyl-hydroxylase domain (PHD) protein. Hydroxylation is required for binding of the von Hippel-Lindau protein (VHL), the recognition subunit of a ubiquitin protein ligase that targets HIF-2α for ubiquitination and proteasomal degradation. In addition, hydroxylation on asparagine residue 847 by factor-inhibiting HIF (FIH) blocks the binding of the co-activator p300. On the other hand, binding of eIF3e/Int6 to the Int6/eIF3e binding site (IBS) leads to the proteasomal degradation of HIF-2α, irrespective of hypoxia/normoxia. This mechanism of post-transcriptional regulation is specific for the HIF-2α subtype. Thus, inhibition of eIF3e/Int6 by siRNA leads to the accumulation of HIF-2α.

Using a yeast two-hybrid approach, Chen et al. identified Int6/eIF3e as a novel regulator of HIF-2α (27). Subtype-specific binding of Int6/eIF3e to HIF-2α at the Int6 binding site (IBS) led to HIF-2α degradation via the proteasome pathway in a hypoxia-independent manner (Figure 1). When specific siRNAs against Int6/eIF3e were used, HIF-2α activity was stabilized even under normoxic conditions, and the expression of several angiogenic factors, such as ANG-1, basic fibroblast growth factor (bFGF), and VEGF, subsequently increased in HeLa and MCF-7 cells (27). The authors extended their investigation of *eIF3e/Int6* silencing to *in vivo* angiogenesis. Injection of siRNA-Int6 into the subcutaneous tissues of mice promoted neoangiogenesis in a dose-dependent manner (94). Additionally, subcutaneous fibroblasts were identified as the main target of the eIF3e/Int6 silencing effects. Subcutaneous *ex vivo* transplantation of siRNA-Int6-transfected fibroblasts induced potent angiogenesis in nude mice. Moreover, co-injection of siRNA-HIF-2α into mouse skin abolished the neoangiogenesis induced by siRNA-Int6, confirming that siRNA-Int6 induced neoangiogenesis and enhanced wound healing by upregulating HIF-2α. Promoter analysis showed that HIF-2α regulated *Int6/eIF3e* and *HIF-2*α expression; Int6/eIF3e, as a negative regulator of HIF-2α stability, reduced HIF-2α protein levels. *Int6/eIF3e* silencing at a certain level inactivated existing and *de novo*-transcribed Int6/eIF3e, reducing the degradation of HIF-2α. The accumulated HIF-2α then further activated *HIF-2*α transcription, and the enhanced accumulation of HIF-2α led to potent angiogenesis. Thus, the effect of Int6/eIF3e silencing on angiogenesis is stronger than that of HIF-2α overexpression. In this way, Int6/eIF3e acts as a master switch of angiogenesis by controlling HIF-2α protein levels in an oxygen-independent manner. *Int6/eIF3e* silencing is an effective way to promote HIF-2α activity in the absence of hypoxia, leading to physiological and functional neoangiogenesis in mice.

## Pathophysiological Roles of HIFs in Angiogenesis and Vascular Remodeling

### Cancer angiogenesis

Oxygen tension is markedly below physiological levels in solid tumors (95, 96). In fact, solid tumors contain severely hypoxic regions, in which pO_2_ values are <10 mmHg (97, 98). Rapidly proliferating cancer cells can outgrow their vascular network, limiting O_2_ diffusion within the tumor itself. Perfusion defects, resulting from abnormal tumor blood vessel structure and function, can also cause hypoxic stress. Consequently, in the tumor tissues of most human cancers, HIF levels are higher than in normal tissues (10, 11). Hypoxia-independent mechanisms also increase HIF-α expression in cancer cells. Various alterations, such as VHL mutation in renal cell carcinoma, mutations in the Wnt/β-catenin signaling pathway in colon cancer, and other oncogenic events, have been reported to elicit HIF-α stabilization (99). HIF-1α activates the transcription of genes that are involved in crucial aspects of cancer biology, including angiogenesis, energy metabolism, cell survival, chemotherapy and radiation resistance, invasion, and metastasis (3, 100). The importance of HIF activity in cancer is evidenced by the fact that increased HIF-α expression correlates with poor clinical prognosis in many cancer types (101). A large body of experimental data shows that manipulations that increase HIF-1α expression result in increased tumor growth, vascularization, and metastasis, whereas loss of HIF activity has the opposite effect (14).

Endothelial cells that interact with malignant cells are also essential components of solid tumor angiogenesis. In mediating angiogenesis, HIF has similar effects on endothelial cells in tumor tissues and in non-malignant tissues. However, unlike “normal” blood vessels, the tumor-associated vasculature is leaky, tortuous, and non-contiguous (102). The microenvironment of the solid tumor is typically hypoxic, and hypoxia-induced changes in the expression of angiogenic factors in cancer cells are critical for tumorigenesis. Loss of HIF-1α in the endothelium inhibits blood vessel growth in solid tumors (103). Tumor-associated endothelial cells interact with tumor cells as well as non-malignant stromal cells, such as fibroblasts and infiltrating bone marrow-derived cells. These cell types differ widely in their responses to hypoxic stress and therefore contribute to tumor angiogenesis in different ways. The selective manipulation of the hypoxic stress response in distinct tumor subcompartments might be more effective as an anti-tumor strategy than systemic HIF inhibition.

### Circulatory and vascular system

The circulatory and vascular system is the first functional organ system required for embryonic survival. HIF-1α homozygous knockout mice show embryonic lethality at mid-gestation, with cardiac malformations, vascular regression, and impaired hematopoiesis (1, 104, 105), suggesting that HIFs are essential for embryonic development of the circulatory system. After maturation of the circulatory system, HIFs continue to mediate adaptive responses to hypoxia by regulating local O_2_ delivery through alterations in vascular tone, angiogenesis, and the remodeling and maturation of collateral vessels. On the other hand, HIFs also regulate the O_2_ content of the blood as a systemic response to hypoxia. EPO increases the blood O_2_-carrying capacity and systemic oxygenation. EPO is a representative example of the way in which HIFs upregulate genes to increase O_2_ delivery to tissues.

Many common disease processes impair or co-opt the physiological responses of the circulatory system. HIF-induced pro-angiogenic factors activate vascular remodeling by binding to receptors on endothelial or smooth muscle cells within vessels. Thus, the HIF pathway also contributes to the regulation of pathophysiological vessel remodeling. Initially, the remodeling is “intended” to provide protection from hemodynamic stresses. However, excessive changes through chronic and repeated activation of the HIF pathway can lead to the pathologic remodeling of vessels.

#### Ischemia

##### Myocardial ischemia

Coronary artery disease (CAD) is the leading cause of mortality in the United States, with a prevalence of 12.3% in the population older than 50 years of age (106). The formation and sudden disruption of atherosclerotic plaques in coronary arteries leads to insufficient myocardial perfusion, either chronically or acutely. Malperfusion can lead to sudden death or the development of heart failure.

Remodeling of collateral arteries is a major physiological response to tissue ischemia. When occlusion of a major coronary artery suddenly disrupts blood flow, patients with a greater number of collateral vessels typically have smaller infarctions, leading to better survival. HIF-1α plays an important role in coronary vascularization, which can be a source of collateral blood flow. Resar et al. investigated the association between genetic variation at the *HIF-1*α locus and the branching of coronary arteries (i.e., collaterals), as determined with angiography (107). They presented evidence that a specific polymorphism in *HIF-*α exon 12 was associated with the absence of coronary collaterals in patients with CAD. The genetic variation also affected the clinical presentation of CAD (108, 109). These studies suggest that *HIF-*α is a major genetic modifier in myocardial ischemia in humans.

Another important issue related to myocardial ischemia is ischemic preconditioning. In ischemic preconditioning, short-term ischemia followed by reperfusion triggers adaptations, such as a shift from oxidative to glycolytic metabolism, that promote survival during O_2_ deprivation (110), thereby conferring protection against a subsequent, prolonged episode of ischemia–reperfusion. In “remote ischemic preconditioning,” brief cycles of ischemia and reperfusion in the limbs protect the heart from ischemic injury (111). Recently, Cai et al. demonstrated that HIF-1 activates *IL-10* transcription; experiments using HIF-1α heterozygous mice showed that HIF-1 was required for remote ischemic preconditioning (112). Interestingly, HIF-1α and HIF-2α have distinct spatial expression patterns in a rat model of ischemic heart disease (113), suggesting that the two subtypes have different roles in the response to hypoxic stress.

##### Limb ischemia (peripheral arterial disease)

Peripheral arterial disease is a disorder in which the chronic obstruction or stenosis of arteries due to atherosclerosis leads to the characteristic symptoms of limb ischemia. Characteristic limb pain during exercise is called intermittent claudication. Further progression of the disease leads to severe ischemia that causes rest pain, ulcer, or gangrene in the affected limbs and digits, called critical limb ischemia (CLI). Patients with CLI often have no option but limb amputation. The prevalence of PAD is in the range of 3–10%, increasing to 15–20% in persons over 70 years old; 1–2% of PAD patients develop CLI (114).

Hypoxia-inducible factors trigger neovascularization in tissues under physiologic and pathologic conditions by stimulating the expression of angiogenic growth factors such as VEGF (103). In hindlimb ischemia models in animals, HIF-1α mediates the adaptive responses to ischemia by increasing the production of angiogenic cytokines. These cytokines include VEGF, stromal-derived factor 1, placental growth factor, angiopoietin 1, angiopoietin 2, and platelet-derived growth factor (PDGF) B. Loss-of-function of HIF-1α, due to aging or heterozygous knockdown, impairs the expression of angiogenic cytokines, the mobilization of angiogenic cells, and the recovery of limb perfusion in the ischemic hindlimb of mice (115). Furthermore, delivery of adenovirus encoding constitutively active HIF-1α stimulates perfusion recovery after femoral artery ligation in older or diabetic mice (115). In these studies, the effect of HIF-1α was attributed to the mobilization of circulating angiogenic cells and the local effects of angiogenic factors.

Given that clinical trials using a single angiogenic factor such as VEGF have failed to promote recovery in patients with PAD, targeting HIFs might be a better therapeutic option because HIF is a master switch that coordinately induces a spectrum of angiogenic factors. Translational studies and clinical trials are further discussed in the Section “Clinical Application of HIF Activators and Inhibitors.”

#### Vascular Wall Disease

##### Aneurysm

Abdominal aortic aneurysm (AAA) is mostly asymptomatic; rupture may be the first manifestation of the disease, which is lethal in most cases. The prevalence of AAA is 8.8% in the population older than 65 years of age (116). The arterial wall of aneurysms is hypoxic (117, 118). The intraluminal thrombus often seen in aneurysms might also limit oxygen diffusion to the aortic wall, leading to wall weakening and rupture (117). AAA tissues in humans express HIF-1α, MMP-2, and Ets-1 within smooth muscle cells and inflammatory infiltrate of the tunica media (119). The expression of HIF-1α is significantly higher in aortic aneurysms than in normal arteries, with increased nuclear translocation, implicating HIF-1α in AAA progression (119). Hypoxia alters vascular smooth muscle cell function, inflammatory processes, and MMPs, decreasing the strength of the arterial wall (120, 121). Results of DNA microarray analyses using specimens of AAA from mice (122) and ruptured intracranial aneurysms from human (123) also suggest that transcription factors, including HIF-1α, have key roles in processes in the aneurysmal vessel wall. Recent studies, using samples from patients with AAA in Poland (124), identified polymorphisms in the *HIF-1*α and *VEGF* genes as potential genetic markers that indicate a predisposition to AAA.

##### Vascular malformation

Vascular malformations encompass a wide spectrum of lesions that can involve every part of the body; they can present as an incidental finding or produce life- or limb-threatening complications. If the disease causes complications or esthetic problems, the therapeutic strategy is multidisciplinary, but interventional radiology, including embolization, sclerotherapy, and laser coagulation, is playing an increasingly important role.

Increased activation of the HIF pathway causes aberrant expression of angiogenic factors that contribute to the formation and maintenance of vascular malformations (125). HIF-1α and VEGF are highly expressed in cerebral arteriovenous malformations (126, 127) and dural arteriovenous fistulas (128). In Sturge–Weber Syndrome vessels, immunohistochemical analysis demonstrated that nuclear HIF-1α and HIF-2α levels were markedly elevated (129). HIF-1α is also associated with the disease progression of vascular malformations (130). These observations might aid the development of therapeutic strategies to treat currently incurable vascular lesions.

##### Varicose veins

Varicose veins, a common disease worldwide, are described as tortuous and dilated palpable veins that are more than 3 mm in diameter. The prevalence has been estimated at 25–33% in women and 10–20% in men (131). Predisposition includes family history, female sex, pregnancy, and prolonged standing. However, the precise pathophysiology of varicose veins remains unknown. While recent studies have focused on endothelial cell integrity and function, including adhesion molecules, increasing evidence suggests that hypoxia explains the pathogenesis of varicose veins. Blood stasis can cause hypoxia in the vein wall (132), and increased expression of HIF-1α in human varicose veins has been reported. In addition, prolonged increases in venous wall tension are associated with overexpression of HIF-1α and HIF-2α, increased MMP expression, and reduced venous contraction in an *ex vivo* animal model of IVC (133). These findings suggest that hypoxia is one cause of varicosity formation (134, 135).

#### Others

##### Atherosclerosis

The rupture of an unstable atherosclerotic plaque in humans causes clinical complications through thrombus formation. These atherosclerotic lesions contain hypoxic areas. Vink et al. reported that HIF-1α was expressed in 49% of carotid and 60% of femoral endarterectomy specimens (4). Another study using human carotid artery specimens from surgery found that HIF-1α expression was higher in atherosclerotic plaques than in control specimens from autopsy (136). The analysis also detected early expression of apoptotic molecules in the atherosclerotic plaque and implicated oxidative stress in triggering inflammatory and apoptotic responses. Emerging evidence suggests that HIF-1α participates in the progression of atherosclerosis by initiating and promoting foam cell formation, endothelial cell dysfunction, apoptosis, inflammation, and angiogenesis (137, 138). Intimal thickening and calcification, which restrict oxygen diffusion into the arterial wall, are thought to contribute to hypoxia in atherosclerotic plaques (139). Plaque inflammation also contributes to hypoxia by increasing oxygen demand (138).

##### Pulmonary arterial hypertension

In chronic lung disease, persistent alveolar hypoxia induces HIF-1α expression in pulmonary arterial smooth muscle cells, resulting in remodeling of the pulmonary vessel and contributing to the pathogenesis of pulmonary arterial hypertension (PAH) and right ventricular dysfunction. The expression of multiple HIF-1α target genes, including endothelin 1 (*EDN1*) (140, 141), transient receptor potential canonical proteins (*TRPC1* and *TRPC6*) (142), and sodium-hydrogen exchanger 1 (*NHE1*) (143), has been reported. Signal transductions results in vasoconstriction and medial thickening, reducing the luminal diameter of pulmonary arterioles and increasing the resistance to blood flow. The fact that mice with heterozygous deficiency in *HIF-1*α and *HIF-2*α are protected from hypoxic pulmonary hypertension indicates that HIF-1α and HIF-2α play pathogenic roles (144, 145).

##### Graft failure

Vascular graft failure after bypass surgery for ischemic disease or after hemodialysis access creation for end-stage renal disease results primarily from stenosis caused by intimal hyperplasia. The mechanism of intimal hyperplasia initiation and development is likely multifactorial, involving endothelial injury and ischemia secondary to tissue handling during the procedure, as well as hemodynamic factors, including hypoxia, shear stress, and mechanical strain (139). Alterations in the HIF pathway might contribute to vascular graft failure through the formation of intimal hyperplasia. Increased hypoxia within the vessel wall in regions of intimal hyperplasia has been observed in animal models with prosthetic grafts (146). At a cellular level, HIF-1α regulates the expression of many genes that are increased in venous neointimal hyperplasia formation, including those encoding macrophage migration inhibition factor, MMPs, and tissue inhibitors of metalloproteinases (147).

##### Venous thromboembolism

Venous thromboembolism is a disease entity comprising deep vein thrombosis, typically in a lower extremity, and pulmonary embolism. Pulmonary embolism presents with a variety of non-specific symptoms, but the onset of pulmonary embolism can lead to sudden death. On the other hand, deep vein thrombosis of the limbs can cause chronic symptoms, referred to as post-thrombotic syndrome. Anticoagulant therapy is the mainstay for the treatment of venous thromboembolism, although surgical treatment or endovascular intervention is an option. Activation of HIF-1α might contribute to the formation (148, 149) and resolution (150–152) of thrombus in this disease entity. Experimental data suggest that stasis of venous blood flow induces localized hypoxemia within the valvular sinus, which is also the predilection site of venous thrombus (153). Hypoxia associated with blood stasis is thought to activate several hypoxia-adaptive responses, including the HIF and early growth response-1 pathways (148).

## Clinical Application of HIF Activators and Inhibitors

Targeting the HIF pathway with pharmacologic agents or gene therapy is a promising therapeutic strategy for the management of various diseases associated with alterations in the HIF pathway. Increased understanding of HIF biology has translated into clinical applications. HIF-modulatory drugs are being developed for diverse diseases. In particular, the therapeutic manipulation of angiogenesis holds great promise for treating diverse pathological conditions, including cancer, macular degeneration, atherosclerosis, and PAD. In this section, pharmacological agents that induce HIF activity are described. Several strategies to promote HIF-1α or HIF-2α activity are in development for use in therapeutic angiogenesis for ischemic diseases. The interventions could be applied to other ischemic injuries such as wound healing.

### PHD inhibitors

In clinical applications, the most advanced pharmaceuticals developed to target the HIF pathway are PHD inhibitors. PHD enzymes are oxygen sensors that act gatekeepers of the adaptive response to hypoxia (99). The oral PHD inhibitors FG-2216 and FG-4592 are being evaluated in clinical trials for the treatment of renal anemia.

Prolyl-hydroxylase domain inhibition, which permits the activation of hypoxic adaptation under normoxic conditions, improves wound healing in diabetic mice (154) and histological and functional outcomes in ischemic and hemorrhagic stroke models (155). Watanabe et al. reported that PHD inhibition after cobalt chloride administration attenuated aneurysm formation in a mouse model of AAA; the effect was associated with a reduction in inflammatory cytokines and in the activity of MMP-2 and MMP-9 (156).

In limb ischemia, Loinard et al. tested whether inhibition of PHDs using small hairpin RNA (shRNA) promoted neovascularization after femoral artery ligation in mice (157). shRNA targeting PHD2 or PHD3 increased vascularization in aged mice through the transient and local upregulation of endogenous HIF-1α.

The oral PHD inhibitor GSK1278863 was tested in a clinical trial in PAD patients with intermittent claudication. However, the trial failed to show a benefit of this compound in regimens of a single dose or a daily dose for 2 weeks (Table 1) (21).

**Table 1:** **Clinical trials for PAD with HIF activators**.

Phase	Condition	Drug	Administration	Results	Reference
1	CLI (critical limb ischemia)	Adenoviral activation of HIF-1α	Intramuscular injection	Well tolerated	(19)
2	PAD (intermittent claudication)	Adenoviral activation of HIF-1α	Intramuscular injection	No benefit	(20)
3	PAD (intermittent claudication)	PHD inhibitor (protein)	Oral	No benefit	(21)

*CLI, critical limb ischemia; HIF, hypoxia-inducible factor; PAD, peripheral arterial disease; PHD, prolyl-hydroxylase domain*.

### Activation of HIF-1α by gene expression

Viral delivery of a constitutively active form of HIF-1α improved the recovery of limb perfusion in ischemic models of aged mice, diabetic mice, and rabbits (108, 115, 158). Based on these results, the first clinical trial using HIF-1α replacement gene therapy was tested in PAD patients with CLI from 1999 to 2004 (Table 1) (19). A recombinant adenovirus, encoding the HIF-1α bHLH-PAS domain fused to the herpes simplex virus VP16 transactivator protein, was administered to 34 patients with no options for surgical and endovascular revascularization. In a phase I study that mainly tested safety, a single intramuscular injection resulted in no serious toxicity, including no evidence of malignancy or ocular neovascularization disorders related to the transgene in the 1-year follow-up. Although the authors noted some favorable clinical responses, the higher death rate and amputation rate in the treatment population prevented further assessment.

Creager et al. tested the same method in a different PAD subpopulation, patients with intermittent claudication. In the prospective, randomized, double-blinded, placebo-controlled, multicenter study from 2005 to 2010, the authors hypothesized that a single intramuscular injection of Ad2/HIF-1α/VP16 would improve peak walking time. However, the hypothesis was not upheld in an assessment of 273 patients who participated in a treadmill exercise test after randomization (Table 1) (20). The possible reasons for the negative result include a low efficacy of gene transfer, an insufficient duration of effect after a single administration, and a lack of functional collateral vessel formation owing to the distance between injection sites.

### HIF-2α stabilization by *Int6/eIF3e* silencing

**Figure 2:**
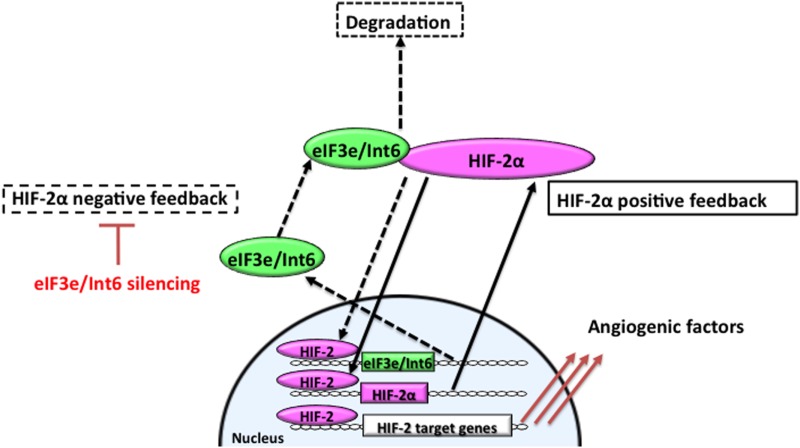
**Feedback mechanisms regulating the expression of HIF-2α**. In hypoxia, stabilized and dimerized HIF-2α recognizes hypoxia responsive elements (HREs) in its own promoter and in the *eIF3e/Int6* promoter, resulting in the transcription of both genes as part of positive and negative feedback mechanisms, respectively. eIF3e/Int6 binds to and degrades newly synthesized HIF-2α, even under hypoxic conditions.

Hypoxia-inducible factor-2α is involved in microvessel remodeling and mature vessel formation (63, 159). Stabilization of HIF-2α through *Int6/eIF3e* silencing promotes functional vessel formation and facilitates the recovery of peripheral circulation and limb function in a hindlimb ischemia model. *Int6/eIF3e* silencing with shRNA delays HIF-2α degradation; stabilized HIF-2α then binds to the HRE in the *HIF-2*α promoter region to upregulate its own expression (Figure 2). On the other hand, because *eIF3e/Int6* has an HRE, the method suppresses the negative feedback loop regulating HIF-2α protein stability. HIF-2α activity increases, as does the downstream expression of a spectrum of angiogenic factors, including VEGF, bFGF, PDGF-B, angiopoietins, and Tie-2 (94). Chen et al. reported that *Int6/eIF3e* silencing through siRNA plasmid injection resulted in a twofold increase in the expression of HIF-2α, without affecting HIF-1α. The treatment enhanced subcutaneous neovascularization and accelerated wound healing in diabetic mice (94). The increase in neovascularization was completely abolished by the simultaneous silencing of *HIF-2*α, suggesting that the improvements depended on HIF-2α. In this model, HIF-2α stimulated vascular sprouting and stabilization.

**Figure 3:**
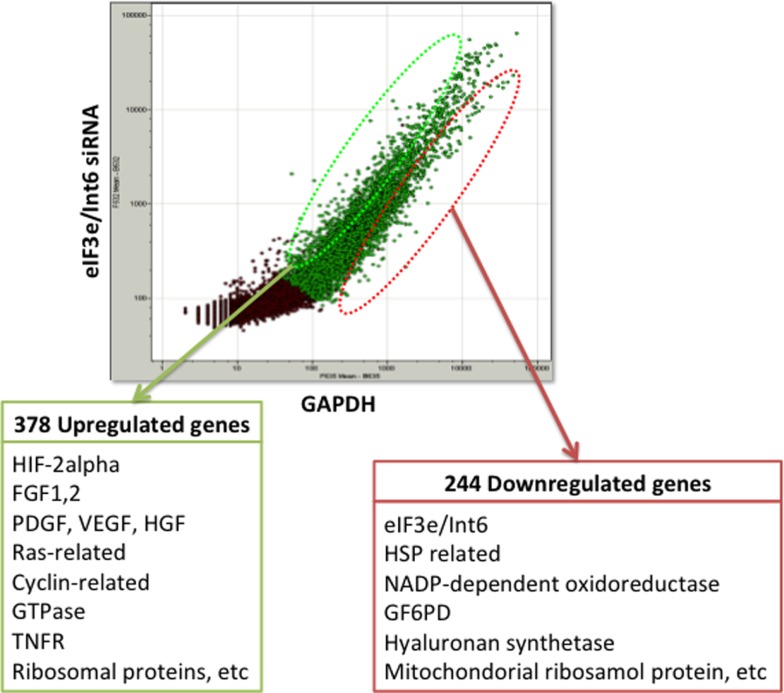
**DNA microarray analysis of *eIF3e/Int6* silencing in MCF-7 cells**. DNA microarray analysis of MCF-7 cells transfected with *eIF3e/Int6* silencing plasmids identified 378 upregulated genes and 244 downregulated genes, relative to expression in cells transfected with *GAPDH*.

The usefulness of *eIF3e/Int6* silencing is also evidenced by several studies using ischemia models. We showed that silencing *Int6/eIF3e* in ischemic thigh muscle enhanced the recovery of peripheral circulation and limb function in a mouse model of femoral artery ligation (160). *Int6/eIF3e* silencing enhanced *PDGF-B* and *bFGF* transcription in muscle cells and the secretion of bFGF and ANG-1, thereby inducing tube formation by endothelial cells via paracrine signaling. *Int6/eIF3e* silencing also decreased brain damage in a rat model of cold injury in the brain, suggesting a potential clinical application for the treatment of brain ischemia and injury (161). Endler et al. have suggested that IL-6 and IL-8 are the main cytokines controlled by the HIF-2α-mediated angiogenic response in endothelial cells (162). To elucidate the mechanism of the strong angiogenic effect of *Int6/eIF3e* silencing, we performed DNA microarray analysis in MCF-7 cells and identified 378 upregulated genes and 244 downregulated genes (Figure 3). The upregulated genes included those encoding the potent angiogenic factors bFGF, PDGF, HGF, and VEGF. These findings indicate that Int6/eIF3e functions as an angiogenic master switch. HIF-2α stabilization by *Int6/eIF3e* silencing might be a promising methodology in clinical practice for the treatment of ischemic diseases such as CAD, cerebral infarction, and PAD. One concern with the method is that the misregulated expression of several eIF3 subunits has been implicated in oncogenesis and in the maintenance of the cancerous state (163). However, we have not observed cancer formation in animal models, including non-human primates.

### Combination treatment with cell therapy

For treatment of limb ischemia, Rey et al. used a two-stage therapy consisting of intramuscular injection of AdCA5 followed 24 h later by intravenous administration of bone marrow-derived angiogenic cells (BMDACs), which were cultured for 4 days in the presence of angiogenic growth factors and dimethyloxalylglycine (DMOG), a hypoxia-mimicking reagent (164). The strategy, which combined HIF-1α gene therapy and cell therapy, improved perfusion and clinical symptoms in a mouse model of CLI. The rationale for the unique staged approach with local and systemic delivery was as follows. Local administration of AdCA5 induces the production of angiogenic factors and thereby provides a homing signal for BMDACs. Systemic administration selects for a subpopulation of cells that migrate to the ischemic tissue and participate in the vascular remodeling process. In contrast, direct injection increases cell death in hypoxic muscle (55). Another recent study found that PHD2 mRNA levels were upregulated in blood cells from patients with CLI, whereas HIF-1α mRNAs levels were attenuated. The study confirmed that PHD2 inhibition enhances the therapeutic potential of cell-based therapy in a CLI mouse model. Cell therapy through modification of the HIF signal might be a potent and promising strategy for the treatment of ischemic disease.

## Future Perspectives

Several hurdles must be overcome if HIF pathway modulation is to be used for therapeutic angiogenesis in clinical settings. First, we must choose the best modality for treatment. We think that an ideal clinical therapy would use intramuscular injection of naked plasmid DNA rather than viral transfer for safety and simplicity, despite the relatively low transfection efficiency of the former approach. To restore blood flow and salvage limbs, cell-based interventions are another promising option for therapeutic angiogenesis in patients with CLI (165). We are investigating a method for cell therapy that uses cultured fibroblasts harvested from the patient’s skin tissue and transfected with an *eIF3e/Int6* silencing plasmid. Second, optimization of drug delivery is important for efficient treatment. Therapeutic site selection is another important factor for the successful development of collateral vessels (166). Further understanding of the HIF pathways will provide insight into the mechanisms responsible for the pathology of various diseases and will facilitate the development of promising therapies aimed at modulating HIF pathways.

## Concluding Remarks

In this review, we summarized the current understanding of the association between HIFs and pathophysiology in the human circulatory system. We also described the regulation of HIFs. Modulation of the HIF system is a potential approach for treating patients who suffer from ischemic diseases. However, for clinical applications, many questions remain to be solved, and greater understanding of the oxygen-dependent and -independent mechanisms that regulate the HIF-α subunits is needed. We must also be cautious about possible side effects. Further translational research and clinical trials for each pathophysiology are warranted.

## Author Contributions

Conception and design: TH, FS; Drafting: TH; Critical revision: FS; Final approval: FS; Overall responsibility: TH, FS.

## Conflict of Interest Statement

The authors declare that the research was conducted in the absence of any commercial or financial relationships that could be construed as a potential conflict of interest.

## Abbreviations

ARNT, aryl hydrocarbon receptor nuclear translocator; bFGF, basic fibroblast growth factor; CAD, coronary artery disease; CLI, critical limb ischemia; eIF, eukaryotic initiation factor; FIH, factor-inhibiting HIF; HAF, hypoxia-associated factor; HDAC, histone deacetylase; HIF, hypoxia-inducible factor; HRE, hypoxia responsive element; IBS, Int6 binding site; IC, intermittent claudication; ID, inhibitory domain; MMP, matrix metalloproteinase; ODD, oxygen-dependent degradation domain; PAD, peripheral arterial disease; PAS, Per–ARNT–Sim; PHD, prolyl-hydroxylase domain; SUMO, small ubiquitin-related modifier; TAD, terminal activation domain; VEGF, vascular endothelial growth factor; VHL, von Hippel-Lindau.
